# Identification of novel surfactin derivatives from NRPS modification of *Bacillus subtilis* and its antifungal activity against*Fusarium moniliforme*

**DOI:** 10.1186/s12866-016-0645-3

**Published:** 2016-03-09

**Authors:** Jian Jiang, Ling Gao, Xiaomei Bie, Zhaoxin Lu, Hongxia Liu, Chong Zhang, Fengxia Lu, Haizhen Zhao

**Affiliations:** College of Food Science and Technology, Nanjing Agricultural University, Key Laboratory of Food Processing and Quality Control, Ministry of Agriculture of China, 1 Weigang, Nanjing, 210095 P.R. China

**Keywords:** Surfactin, NRPS, Module deletion, *Fusarium moniliforme*

## Abstract

**Background:**

*Bacillus subtilis* strain PB2-L1 produces the lipopeptide surfactin, a highly potent biosurfactant synthesized by a large multimodular nonribosomal peptide synthetase (NRPS). In the present study, the modules SrfA-A-Leu, SrfA-B-Asp, and SrfA-B-Leu from surfactin NRPS in *B. subtilis* BP2-L1 were successfully knocked-out using a temperature-sensitive plasmid, pKS2-mediated-based, homologous, recombination method.

**Results:**

Three novel surfactin products were produced, individually lacking amino acid Leu-3, Asp-5, or Leu-6. These surfactins were detected, isolated, and characterized by HPLC and LC-FTICR-MS/MS. In comparison with native surfactin, [∆Leu^3^]surfactin and [∆Leu^6^]surfactin showed evidence of reduced toxicity, while [∆Asp^5^]surfactin showed stronger inhibition than native surfactin against *B. pumilus* and *Micrococcus luteus*. These results showed that the minimum inhibitory concentration of [∆Leu^6^]surfactin for *Fusarium moniliforme* was 50 μg/mL, such that [∆Leu^6^]surfactin could lead to mycelium projection, cell damage, and leakage of nucleic acids and protein. These factors all contributed to stimulating apoptosis in *F. moniliforme*.

**Conclusions:**

The present results revealed that [∆Leu^6^]surfactin showed a significant antifungal activity against *F. moniliforme* and might successfully be employed to control fungal food contamination and improve food safety.

## Background

*Fusarium moniliforme* mainly contaminates maize, sorghum, wheat, cotton, beans, tomatoes, peanuts, bananas, beans, peppers, and some feeds. Among these materials, maize is the most prone to fungal infection, accounting for almost 90 % of all types of food pollution [1, 2]. As one of the most common fungi, *Fusarium* mycotoxin researchers are currently most concerned about *F. moniliforme*. Currently, surfactins are used for their antibacterial, antiviral, anti-tumor, and hemolytic activities [3–6]. However, surfactins do not only inhibit filamentous fungi, but C15 surfactin has a synergistic inhibition effect on filamentous fungi. The lipopeptide surfactin family has a ring structure peptide chain and possesses a β-hydroxy fatty acid chain (typically C_13_–C_16_) containing seven amino acids formed by crosslinking [7]. There has been great interest in these compounds because of their potential biological activities as well as economic value. Lipopeptides are often composed of seven or fewer modules composed of amino acids components. Surfactin consists of a Glu-Leu-Leu-Val-Asp-Leu-Leu peptide, synthesized by large multifunctional nonribosomal peptide synthetases (NRPSs) via the multiple thiotemplate mechanism [8, 9]. The composite module can be modified by epimerization, methylation, acylation, or cyclization. The final lipopeptide products can have linear, cyclic, or branched peptide backbones [10].

In this study, a procedure is described that allows for efficient and relatively fast inactivation of a *Bacillus subtilis* gene to create new, biotechnologically interesting products. The approach is the same as developed has been for some other Gram-positive strains [11, 12] and uses a high temperature-sensitive, shuttle plasmid based on the pKS2 replication origin. Plasmid pKS2 replicates at 30 °C, but 37 °C is nonpermissive for plasmid replication. This method is different from the traditional two-step knockout method [13] and can quickly knock out a module with precision. In contrast, the two-step method usually cannot avoid the impact of an exogenous antibiotic resistance gene.

*B. subtilis* strain BP2-L1 produces surfactin following the integration of genes *sfp* and *degQ* into the *B. subtilis* BP2 chromosome [14]. For knock out of the modules SrfA-A-Leu, SrfA-B-Asp, and SrfA-B-Leu of surfactin NRPSs in *B. subtilis* BP2-L1, the pKS2-mediated, temperature-sensitive, homologous recombination method was used. The structures of the resulting novel surfactins were identified and isolated to develop new antibacterial lipopeptides with stronger antimicrobial activity and more beneficial characteristics.

## Methods

### Strains, plasmids, and media

Strains and plasmids used in this study are listed in Table 1. *B. subtilis* strain PB2-L1, a derivative of *B. subtilis* 168 (trpC2) developed to produce surfactin [14], was used as the source of surfactin synthetase genes and for engineering surfactin synthetase. *B. subtilis* PB2 was a model strain of *Bacillus subtilis* from Chester Price’ lab of UCDavis. pMD19T-simple vector was a commercial carrier and pKS2 vector was temperature sensitive vector for gene deletion. *Escherichia coli* DH5α was used for cloning procedures and propagation of plasmids; pKS2-based vectors can be replicated at 30 °C in *E. coli*. Before transforming *B. subtilis*, plasmids were purified from *E. coli* strain JM110 to obtain the unmethylated forms. Bacterial cells were cultivated in Luria broth (LB, 5 g yeast extract/L, 10 g peptone/L, and 10 g NaCl/L) or in Landy medium [15] supplemented with 0.1 % yeast extract and 2 mg/L phenylalanine [16], at temperatures of 28 or 37 °C. Ampicillin was added to 100 μg/mL.

**Table 1:** Bacterial strains and plasmids used in this study

Strain/plasmid	Relevant genotype/description	Reference
*E. coli*		
DH5α	recA1, endA1, lacZDM15	New England Biolabs
JM110	F',traD36proA^+^B^+^lacI^q^lacZΔM15/damdcmsupE44hsdR17 thi leu thr rpsL lacY galK galT ara tonA tsx∆ (lac-proAB)	Transgen Biolabs
*B. subtilis*		
*B. subtilis* PB2	*B. subtilis* 168 trpC2	Chester Price’ lab (UCDavis, USA)
*B. subtilis* PB2-L1	Derivative of *B. subtilis* PB2 Produces surfactin	Our labs
*B. subtilis* LS1	Lacking the third D-leucine module from *B. subtilis* PB2-L1	This work
*B. subtilis* LS6	Lacking the fifth L-aspartate module from *B. subtilis* PB2-L1	This work
*B. subtilis* LS9	Lacking the sixth D-leucine module from *B. subtilis* PB2-L1	This work
Plasmids		
pMD19T-simple	TA cloning vector; Amp^R^	TAKARA
pKS2	Thermosensitive vector; Kan^R^,Erm^R^	Our labs
pKS2-srfA-C-∆Leu	Third D-leucine module knock-out vector; Kan^R^,Erm^R^	This work
pKS2-srfA-B-∆Asp	Fifth L-aspartate module knock-out vector; Kan^R^,Erm^R^	This work
pKS2-srfA-B-∆Leu	Sixth D-leucine module knock-out vector; Kan^R^,Erm^R^	This work

### Plasmid construction

The 0.59-kb fragment of the upstream SrfA-A-Leu module and 0.51-kb fragment of the downstream SrfA-A-Leu module were amplified using the primer pairs, 5′srfA-A-∆Leu-up-F/3′srfA-A-∆Leu–SOE-up-R and 5′srfA-A-∆Leu–SOE-down-F/3′srfA-A-∆Leu-down-R, respectively. Because of the 15 bp overlapping fragment in 3′srfA-A-∆Leu–SOE-up-R and 5′srfA-A-∆Leu–SOE-down-F, these two fragments were used as templates for overlapping PCR with the primers 5′srfA-A-∆Leu-up-F and 3′srfA-A-∆Leu-down-R [17]. The 1107 bp upstream and downstream fragments of SrfA-A-Leu module were modified with KpnI and XhoI and ligated with similarly treated *E. coli* and *B. subtilis* shuttle vector pKS2 to yield pKS2-srfA-C-∆Leu (Table 1). The construction of pKS2-srfA-B-∆Asp and pKS2-srfA-B-∆Leu used similar methods. The Accession Numbers of all nucleic acid primers is NC_000964.3 from NCBI database.

### *B. subtilis* strain construction

Traditional chemical transformation was used in *B. subtilis* strain construction. The genotypes of new transformants were identified via PCR. *B. subtilis* PB2-L1 transformed with the temperature-sensitive vectors pKS2-srfA-C-∆Leu, pKS2-srfA-B-∆Asp, and pKS2-srfA-B-∆Leu. The host strain *E. coli* JM110 can modify the shuttle vector pKS2 by demethylation and, by modifying demethylation, the rate of *B. subtilis* transformation can be highly improved. New transformants possess erythromycin resistance, such that these transformants were selected on LB medium agar plates with 10 μg/mL erythromycin [18].

**Fig. 1:**
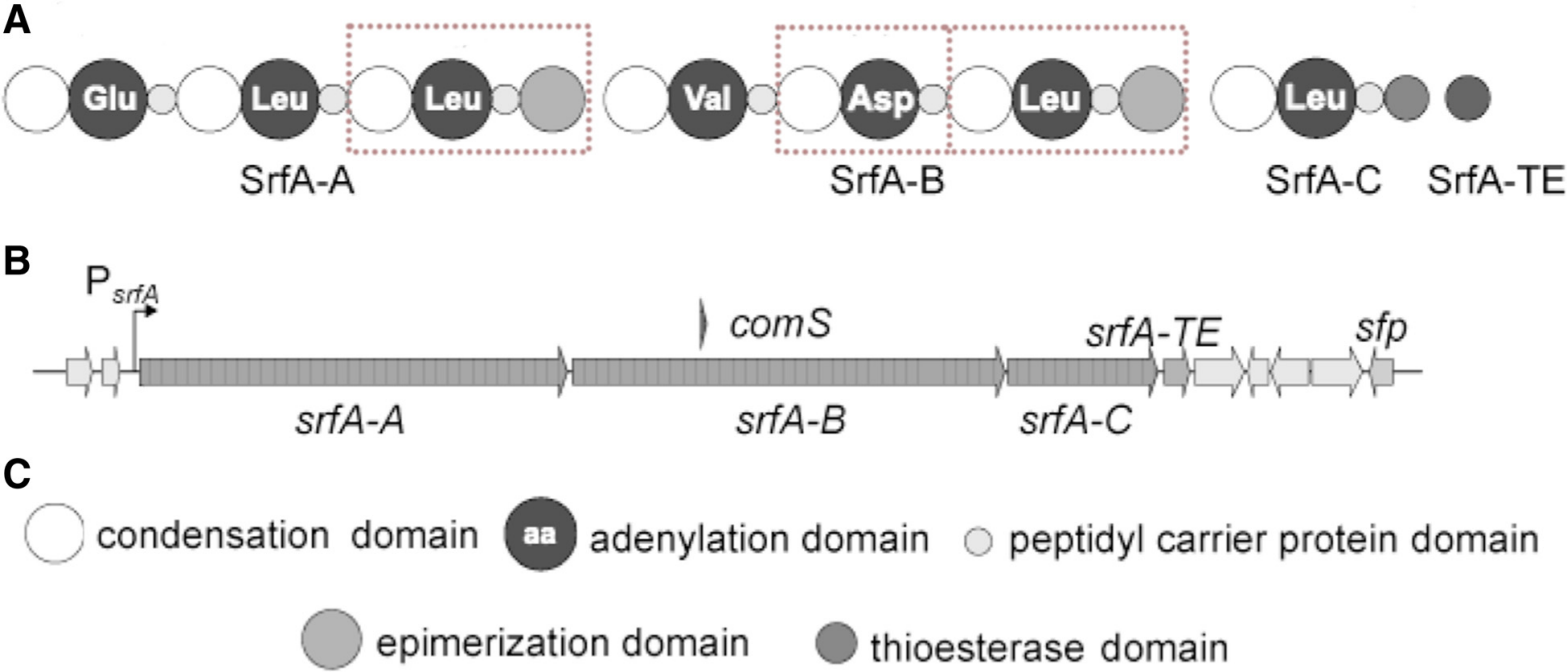
the surfactin A biosynthesis gene cluster. The surfactin synthetases A is composed of SrfA-A, SrfA-B, SrfA-C and SrfA-TE, respectively (**a**). The assembly line of surfactin in the genome consists of three polycistronic genes srfA-ABC (**b**) which can be further subdivided into five functional domains (**c**). The dotted boxes indicate the three modules we deleted in this work

Surfactin is a lipopeptide of seven modules that are assembled by NRPS A-, PCP-, C-, and modifying domains (Fig. 1). This antibacterial lipopeptide must be linearly arranged, synthesized, and cyclized into the final assembly of seven amino acids and a β-hydroxy fatty acid chain. Knocking out one of the modules in NRPS gene clusters produces a lipopeptide lacking one amino acid.

**Fig. 2:**
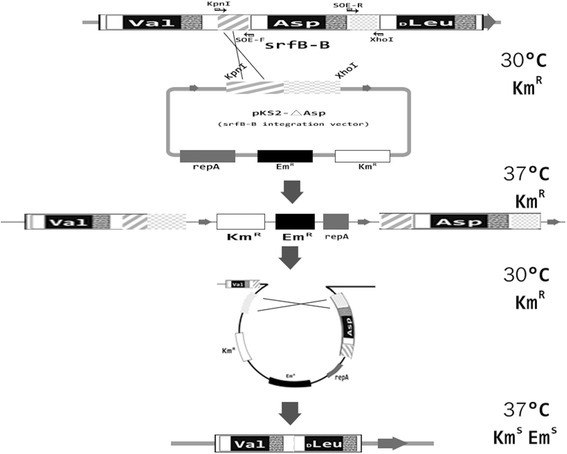
Knockout strategy of marker-free gene. Note that recombination may occur, both at the upstream fragment of the target gene, as shown below, and at the downstream fragment. In both cases, the final connection result of the chromosome is the same thing. All of intermediation process was identified by polymerase chain reaction

Gene exchange is a two-step replacement procedure, as described previously [19]. Resistance genes were replaced in two steps and recombinant strains sensitive to antibiotics (Fig. 2). In the first step, the gene recombinant plasmid from *B. subtilis* strains was cultured in LB medium at 37 °C. The homologous sequences were within the target gene plasmid and the entire plasmid inserted into the genome via a single crossover. In Fig. 2, crossed lines indicate the position of a single crossover and the diagonal block and little dots indicate homologous fragment positions. In the second step, the above-described integrands took place in a single exchange; the second exchange occurred on the chromosome in a parental or homologous sequence at 30 °C within 48 h and finally expelled the plasmid [20]. In Fig. 2, the diagonal block on the plasmid and genome was successfully integrated by the initial and second single crossovers, such that the little dots in the block occurred between the plasmid and genome. Colonies with deleted surfactin modules exhibited the erythromycin sensitive phenotype, which was then used to detect the desired genotype. Finally, sensitive clones were obtained and verified by PCR. These erythromycin gene of knockout mutants could not amplify this resistance gene but could amplify upstream and downstream sequences of homologous gene knockout mutants (amplified sequence could not contain the knockout gene sequence).

### Culture conditions for obtaining surfactins

*B. subtilis* strains were inoculated into 250-mL flasks containing 100 mL of LB medium and cultured at 37 °C for 24 h with 180 rpm shaking as a preculture. A 5 % (by vol) preculture was inoculated into a 500-mL flask containing 200 mL of Landy medium [15], and the wild-type and modified surfactin produced by culturing at 30 °C with 180 rpm shaking for 72 h.

### Extraction of a novel surfactin

After cultivation, a culture was centrifuged at 11000 × g for 15 min to remove bacterial cells. The supernatant pH was adjusted to 2.0 by adding 6 N HCL until the supernatant produced a precipitate. The supernatant was then centrifuged at 11000 × g for 10 min and the precipitate collected [21]. The precipitate was then resuspended in methanol several times with subsequent centrifugation. Finally, the pH was adjusted to 7.0 using 6 N NaOH to obtain an extract dissolved in methanol.

### Identification of a novel surfactin

A lipopeptide surfactin was extracted as described previously [22]. The extracts were analyzed by HPLC/MS (Hewlett Packard 1100 Series C8 column, Hewlett-Packard Co., Palo Alto, CA, USA) and monitored at 210 nm as well as in negative-ion mode over the *m/z* range from 500 to 1200. The solvent gradient profile used buffers A and B (0.05 % aqueous formic acid and 0.045 % methanolic formic acid, by vol, respectively) at a flow rate of 0.3 mL/min, with sample elution starting with 70 % buffer B, followed by a linear gradient to 100 % buffer B over 30 min.

All samples were analyzed by continuous infusion into the LTQ® 7 Tesla FTICR mass spectrometry ion trap (LTQ-FT, Thermo Fischer Scientific Inc., Waltham, MA, USA) equipped with a Triversa Nanomate nanoESI ion source (Advion Biosciences Corp., Ithaca, NY, USA) [23]. Samples were detected under negative ion mode (mass range, 150–1500) using the following parameters: ion spray voltage at 2.5 kV, sheath gas at 20 units, capillary temperature at 300 °C, capillary voltage at 41 V, and tube lens at 110 V. Xcalibur software was used for visualization of high-resolution spectral profile data (Thermo Fischer Scientific, Inc., 2nd Edition SP2) and the exact patterns of *m/z* values obtained.

### Biological activity analysis of novel surfactin

Surfactin hemolytic activity was analyzed using blood-agar plates [24]. In brief, activity was detected on commercial blood agar using the Oxford Cup for hemolytic activity detection. The ability of products from bacterial strains to inhibit the growth of various indicator organisms by the agar well diffusion method was a qualitative determination [25]. Pre-poured agar media plates were spread with 10^7^ CFU/mL of the respective indicator organism and allowed to dry. Wells of 6.8 mm diameter were cut in the plates using a sterile steel borer and filled with 24 h LB culture filtrate (60 μL) of each isolate. After incubation under appropriate conditions, the diameter of the inhibition zone was measured by using calipers. *B. pumilus* strains were mixed in LB agar plates, and surfactin A and one of three novel surfactin extracts added into the plates using the 6.8-mm punch method to detect lipopeptide antibiotic activity. Filamentous growth of *F. moniliforme* were mixed in potato dextrose agar (PDA) plates, and antibacterial activity measured as described previously. Biologically active substances were isolated and purified by HPLC to yield relatively pure products. Under vacuum conditions, the same concentration ratio and sample concentration were obtained for these isolates and the biological activity analyzed. The diameters of cleared zones were measured and are shown in Table 3. Each sample was treated in triplicate and the results expressed as means ± SD.

According to the National Committee for Clinical Laboratory Standards (NCCLS), the minimum inhibitory concentrations (MICs) of purified novel surfactin were detected by the 96-plate microbroth method [26]. Final concentrations of novel surfactin in mixtures ranged from 12.5 to 800 μg/mL. MICs were measured using an ELISA analyzer after incubation of bacteria at 37 °C for 20 h and the fungus at 28 °C for 24 h.

### Inhibitory ability of [∆Leu^6^]surfactin on *F. moniliforme* hyphae and spores

Equal volumes of 6.25, 12.5, 25, 50, 100, 200, and 400 μg/mL of [∆Leu^6^]surfactin were separately added into PDA medium. Then, 5-mm hyphae discs of *F. moniliforme* were placed at the PDA media centers. The diameters of the hyphal extent were detected by the decussation method after incubation for 7 d at 28 °C and then the inhibition ability rate calculated. At the same time, after washing hyphae of *F. moniliforme* using 10 mL of saline solution containing Tween 20 (0.1 % by vol), the spore numbers were detected using a hemocytometer. A sample with sterile PBS was used as a control and each group was evaluated in triplicate.

### Influence of *F. moniliforme* hyphae by [∆Leu^6^]surfactin

Cells at 1 × 10^6^ cell/mL of *F. moniliforme* were cultured on individual PDA culture plates at 28 °C for 5 d. Mycelia were harvested from cultures and prepared by washing with sterile saline solution (0.85 % NaCl, by wt) and then centrifuged at 3,000 × g for 3 min. Then, hyphae were picked from PDA plates, placed on the concave side, and [∆Leu^6^]surfactin solution added to a final concentration of 50 μg/mL. The hyphae were cultured in moisturizing gauze on Petri dishes and, after incubation for 1, 2, or 4 h at 28 °C, samples were collected for inspection by ordinary optical microscopy. Sterile PBS was used as a control.

### SEM and TEM

Hyphae were washed in 0.1 M phosphate buffered saline (PBS, pH 7.2), and fixed using 2.5 % glutaraldehyde at 4 °C for 24 h. The resulting sediments were rinsed three times with 0.02 M PBS, fixed with 2 % osmium tetraoxide for 2 h at room temperature, and dehydrated through a sequence of 30, 50, 70, and 90 % aqueous ethanol solutions. Morphological observations of samples were carried out using a scanning electron microscope (Hitachi High Technologies America, Inc., Shaumburg, IL, USA Inc.) operating at 30.0 kV.

A modified TEM method was used, as previously described [27]. Hyphae were fixed using 2.5 % glutaraldehyde at 4 °C for 24 h and, after treatment with 2 % osmium tetraoxide, ethanol, acetone, and epoxy, 100-nm-thick specimens were cut using a microtome (HM 505E, Microm GmbH, Walldorf, Germany). Specimens were then observed by TEM (JEM-1230, Jeol Ltd., Tokyo, Japan).

### Spore analysis by flow cytometry

Spore suspensions of *F. moniliforme* at 1 × 10^6^ cell/mL were provided with [∆Leu^6^]surfactin to a final concentration of 12.5, 25, or 50 μg/m and cultured at 28 °C for 1–2 h. After incubation, the spores were collected, centrifuged (5000 × g for 3 min), and washed twice with 0.02 M PBS. The retrieved spores were suspended, adjusted to 1 × 10^6^ cell/mL, dyed by adding 10 μL of 1 mg/mL of propidium iodide solution, and then incubated at 4 °C in darkness for 15 min [28]. A Becton Dickinson FACScalibur (BD Biosciences, Inc., San Jose, CA, USA) was used for flow cytometry analysis and FCS Express 4 software used for data analysis. Counts of 10,000 per sample were obtained and each sample analyzed in triplicate. The percentage of spore cell damage was calculated using the following formula: Cell damage (%) = {(the number of stained cells M2)/(the number of non-stained cells M1 + the number of stained cells M2)} × 100 %.

### Impact of [∆Leu^6^]surfactin on *F. moniliforme* nucleic acids and proteins

Spores of *F. moniliforme* at 1 × 10^6^ cell/mL were treated with 25 and 50 μg/mL of [∆Leu^6^]surfactin and then incubated in PDA medium at 28 °C for 1–4 hours. Spores not treated with [∆Leu^6^]surfactin served as controls. After incubation, the supernatant was retrieved from samples by centrifugation at 5000 × g for 3 min. Finally, the samples’ OD_260_ and OD_280_ levels were measured to assess the leakage of nucleic acids and proteins from *F. moniliforme* [29].

### DNA binding assay by [∆Leu^6^]surfactin

DNA binding was detected by gel retardation experiments, as described previously [30]. *F. moniliforme* DNA was mixed with different concentrations of [∆Leu^6^]surfactin and surfactin with 10 μL of binding buffer (10 mM Tris–HCl and 1 mM EDTA buffer, pH 8.0). The mixed samples were incubated for 1 h and then the mixtures assessed using 1.0 % agarose gel electrophoresis.

### Statistical analysis

Statistical analyses were determined using SPSS software (SPSS version 17.0, IBM Corp., Armonk, NY, USA). All experiments were performed in triplicate and data expressed as mean ± standard deviation (SD). A *p* value of <0.05 was considered significant.

## Results

### Reconstitution of novel surfactin synthetase

The first step in SrfA-A and SrfA-B subunit rearrangement was the deletion of *D*-Leu-, Asp-, and *D*-Leu- modules. Deletion of the SrfA-A-Leu, SrfA-B-Asp, or SrfA-B-Leu modules in the srfA-A or srfA-B subunit was completed using the marker-free method (Fig. 2). In the first deletion, a 4.4 kb fragment of SrfA-A-Leu was deleted with an upstream and downstream integration by means of the temperature sensitive shuttle plasmid pKS2-SrfA-A-∆Leu. The deleted fragment located between 1050 and 2090 (the deleted fragment of srfA-A subunit corresponding to amino acid positions) in the srfA-A subunit. The second deletion mutant was produced using the disruption plasmid pKS2-SrfA-B-∆Asp. This disruption plasmid deleted the SrfA-B-Asp module from position 1196 to 2092, which encoded an *L*-Asp-incorporating module in the srfA-B subunit. The third module mutant introduced a deletion in the SrfA-B-Leu module from position 2093 to 3574, which encoded the *D*-Leu-incorporating module in the srfA-B subunit.

The upstream and downstream sequences of the deleted fragment were amplified using PCR, using the corresponding primers, and the occurrence of homologous recombination demonstrated. The resulting plasmid-less clones were screened for the desired modifications using colony PCR analysis. In deletion mutants, the erythromycin resistance gene in the chromosome was removed at the last step and, thus, the erythromycin resistance gene could not be amplified from the deletion mutants by PCR.

Using amplification primer pairs, 5′srfA-A-ΔLeu-up-F and 3′srfA-A-ΔLeu-down-R, and 5′pKS-1058-ERM-F and 3′pKS-1058-ERM-R (Table 2), the target band was 1107 bp, which described mutants containing the upstream and downstream sequence of the Leu module. PCR validation confirmed that, at the molecular level, the deleted module sequence was indeed not in the mutant genome. The deletion of SrfA-B-Asp and SrfA-B-Leu modules was also in accordance with the above methods.

**Table 2:** Primers used in this study. Underlined sequences are complementary sequences to adjacent segments

Oligonucleotide	Sequence
5’srfA-A-∆Leu-up-F	5’-CAAGATACGTATCCT-3’
3’srfA-A-∆Leu–SOE-up-R	5’-CAGCATTCCCTCCTGAGTCGGAAGCGTCAG-3’
5’srfA-A-∆Leu–SOE-down-F	5’-CTGACGCTTCCGACTCAGGAGGGAATGCTG-3’
3’srfA-A-∆Leu-down-R	5’-CCACTTGATGTAATC-3’
5’srfA-B-∆Asp -up-F	5’-CAGCATTATCCTGTATC-3’
3’srfA-B-∆Asp–SOE-up-F	5’-AGCAGACGCCTCCATTGGCCGCTCGAAATC-3’
5’srfA-B-∆Asp–SOE-down-F	5’-GATTTCGAGCGGCCAATGGAGGCGTCTGCT-3’
3’srfA-B-∆Asp-down-R	5’-TTGCCAAACGGCG-3’
5’srfA-B-∆Leu-up-F	5’-ATGGAGGCGTCTGCT-3’
3’srfA-B-∆Leu–SOE-up-F	5’-GCTAAATTGACTCATTTGCCAAACGGCGAA-3’
5’srfA-B-∆Leu–SOE-down-R	5’-TTCGCCGTTTGGCAAATGAGTCAATTTAGC-3’
3’srfA-B-∆Leu-down-R	5’-CGGCTTTTGTTCGCG-3’
5’pKS-1058-ERM-F	5’-CTTTGGCGTGTTTCATTGCTTG-3’
3’pKS-1058-ERM-R	5’-GGTTCGTGTTCGTGCTGACTTG-3’

### HPLC-MS analysis of novel surfactin production

Culture supernatants were acidified and the resulting precipitates extracted with methanol and analyzed by reverse-phase HPLC. Wild-type surfactin A was detected as a group of four major peaks representing fatty acid length polymorphism. Novel forms of surfactin, [∆Leu^3^]surfactin, [∆Asp^5^]surfactin, and [∆Leu^6^]surfactin, were observed with retention times of 17.3, 18.7, and 17.8 min, respectively. The final yield was ~0.82, 1.35, and 0.96 mg/L, respectively. Because the novel surfactin secretions by strain *B. subtilis* LS1 appeared to be more hydrophobic than native surfactin A, in the LC atlas, the LS6 retention time by comparison was later than the retention time of LS1 and LS9.

The lipopeptide biosyntheses of mutant *B. subtilis* LS1, LS6 and LS9 were compared and HPLC results showed that the novel surfactin yields from these mutant strains were relatively low. Thus, the three novel surfactin structures were analyzed and identified by FTICR-MS, a highly sensitive detection method.

**Fig. 3:**
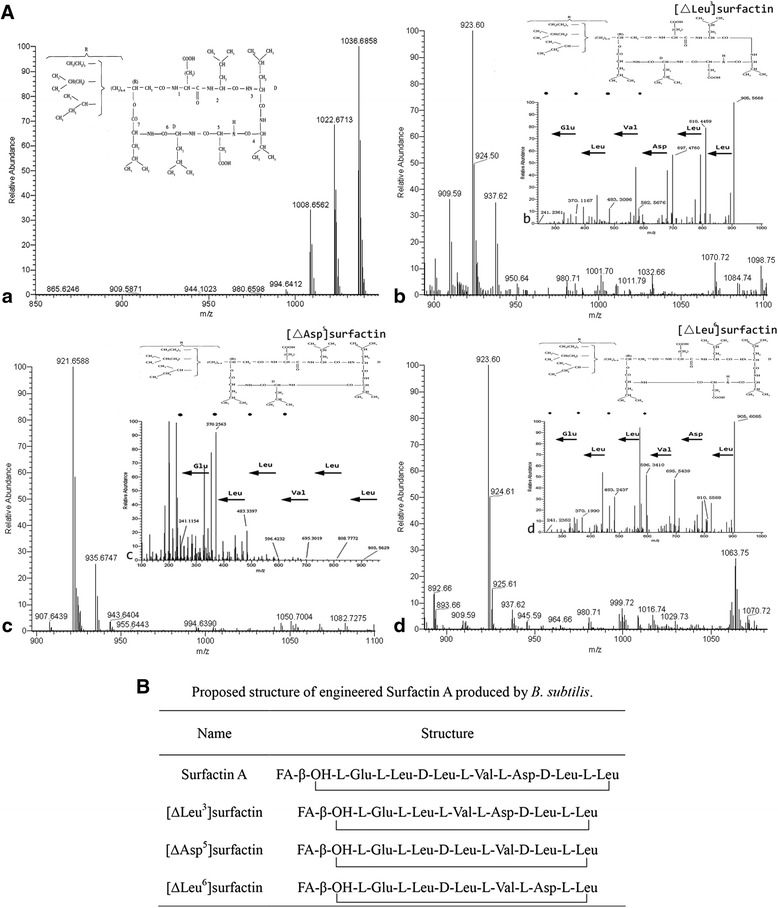
A. FTICR-MS of lipopeptides produced by *B. subtilis*. (A) *B. subtilis* PB2-L1, (B) *B. subtilis* LS1, (C) *B. subtilis* LS6 and (D) *B. subtilis* LS9. The values 1008.7, 1022.7 and 1036.7 correspond to the calculated H^+^ adducts of surfactin A (A) with a fatty acid residue ranging from 13 to 15 carbon atoms. The values 909.6, 923.6 and 937.6 correspond to the calculated H^+^ adducts of [∆Leu^3^]surfactin (B) with fatty acid bodies ranging from 14 to 16 carbon atoms. The values 907.6, 921.7 and 935.7 correspond to the calculated H^+^ adducts of [∆Asp^5^]surfactin (C) with fatty acid bodies ranging from 14 to 16 carbon atoms. The [∆Leu^6^]surfactin values (D) are the same as the [∆Leu^3^]surfactin values (B). The (b, c, d) shows FTICR-MS/MS of [∆Leu^3^]surfactin, [∆Asp^5^]surfactin, [∆Leu^6^]surfactin. B. The proposed structure of engineered Surfactin A produced by *B. subtilis*

The molecular mass of surfactin A in the range *m/z* 1008–1036 was similar to previous published molecular masses (Fig. 3a) [31], and its fatty chain length was 13–15 carbon atoms. The molecular mass of [∆Leu^3^]surfactin was in the range of *m/z* 909–937 (Fig. 3b). The other two novel surfactins, [∆Asp^5^]surfactin and [∆Leu^6^]surfactin, were also confirmed by mass spectra of ions at *m/z* 907–935 and 909–937 (corresponding to H^+^ adducts), respectively (Fig. 3c and d). Overall the chain lengths of these three novel surfactin derivatives were in the range of 14 to 16 carbon atoms, such that surfactin A and the three novel surfactin derivatives showed different chain lengths in their β-hydroxy fatty acids.

**Fig. 4:**
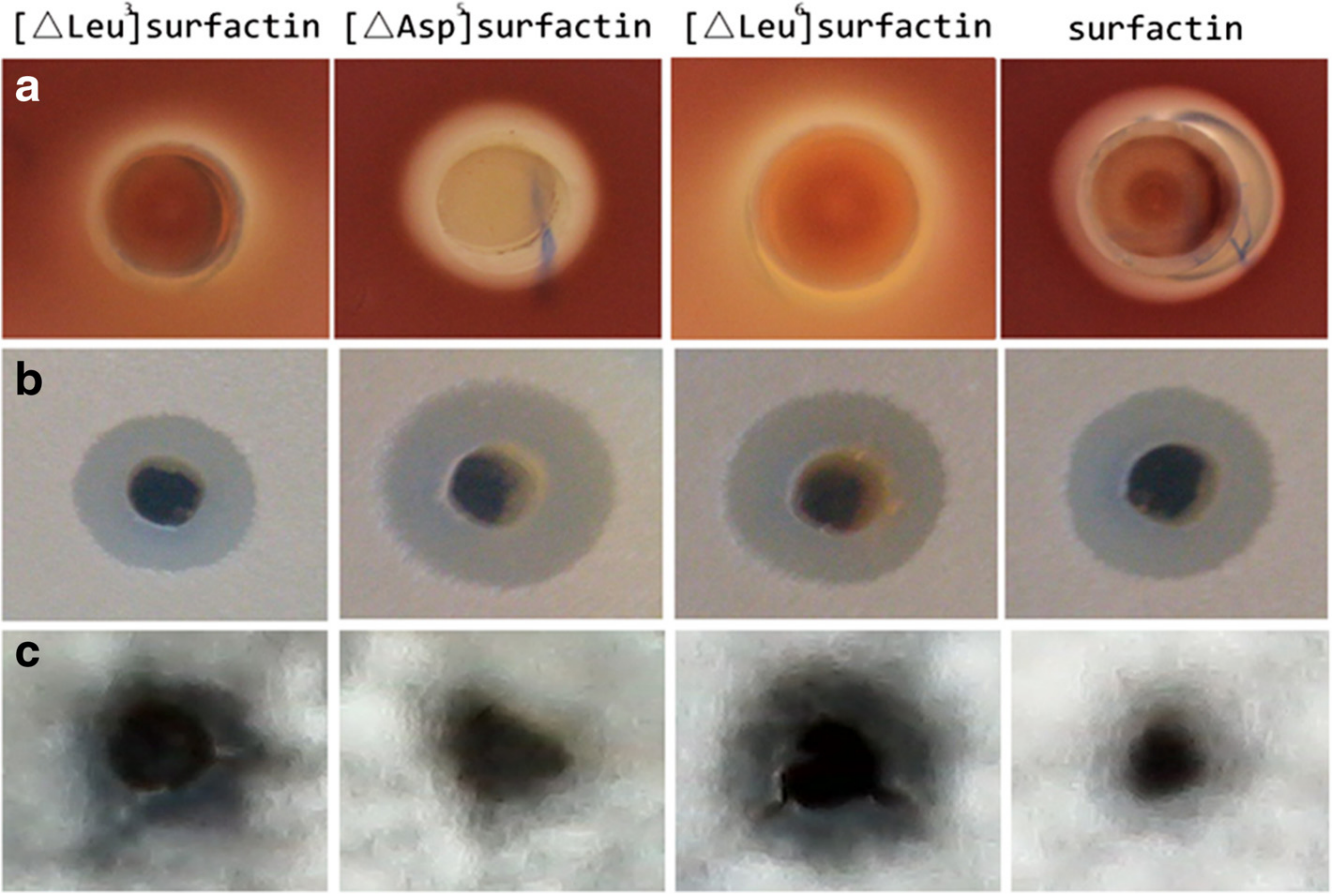
Analysis of bioactivity activity of novel surfactins. **a** The activity of hemolysis after incubation on blood agar plates for 24 h at 37 °C. **b** Inhibition of *Bacillus pumilus* after incubation on LB agar plates for 12 h at 37 °C. (C) Inhibition of *Fusarium moniliform* after incubation on PDA plates for 48 h at 28 °C. **a** was the hemolysis test; (**b**) and (**c**) were the antibacterial and antifungal test

Figure 3b, c, and d show the fracture fragments from [∆Leu^3^]surfactin, [∆Asp^5^]surfactin, and [∆Leu^6^]surfactin. [∆Leu^3^]surfactin corresponded to [M + H]^+^ ions of *m/z* 923.6 Da. A deduction of 18 Da occurred because of dehydration by the FTICR-MS/MS conditions. Thus, the initial molecular weight of [∆Leu^3^]surfactin was [M + H]^+^ ions of *m/z* 905.6. The stepwise cleavage was *L*-leucine (810.4), *D*-leucine (697.5), *L*-aspartic acid (582.6), *L*-valine (483.3), *L*-leucine (370.1), and *L*-glutamic acid (241.2) from [∆Leu^3^]surfactin (Fig. 4b, arrows indicate fragment size and theoretical molecular weight of each amino acid). FTICR-MS/MS results for [∆Asp^5^]surfactin and [∆Leu^6^]surfactin were marked in the same manner as for [∆Leu^3^]surfactin (Fig. 3c and d). FTICR-MS/MS results indicated that fracture fragments of these three novel surfactin were consistent with the predicted patterns.

### Bioactivity analysis of novel surfactins

Lipopeptide surfactin A causes hemolysis and inhibits a broad range of microorganisms, but it does not inhibit filamentous fungi. Blood agar plates and surfactin extracts from *B. subtilis* PB2-L1, LS1, LS6, and LS9 were cocultured at 37 °C for 24 h. Hemolytic activity was detected using the Oxford Cup method. [∆Leu^3^]surfactin produced a clear hemolytic zone surrounding extracts of culture supernatants (Fig. 4a). In contrast, no hemolysis was visible for [∆Leu^3^]surfactin and [∆Leu^6^]surfactin. However, the hemolytic zone of methanol extracts from *B. subtilis* LS6 was bigger than that for *B. subtilis* PB2-L1.

Growth inhibitions of microorganisms by the three novel surfactins were compared by incubating the mixtures at 37 °C for 1 d. The inhibition zone of [∆Asp^5^]surfactin was very obvious, presenting a clear, transparent circle around the hole (Fig. 4b). In contrast, surfactin A caused less fungal inhibition. In fungal inhibition experiments using the three novel surfactin derivatives, [∆Leu^3^]surfactin and [∆Leu^6^]surfactin exhibited the ability to inhibit fungi, and surfactin A and [∆Asp^5^]surfactin did not show such ability. These results indicated that [∆Leu^6^]surfactin and [∆Leu^3^]surfactin possessed inhibitory ability for *F. moniliforme* hyphae, compared with surfactin (Table 3). For example, the colony diameters of *Fusarium moniliforme* were 13.12 ± 0.49 mm for [∆Leu^6^]surfactin, and the surfactin showed no inhibition of *F. moniliforme* hyphae growth.

**Table 3:** Diameter of cleared zones of surfactins exhibiting biological activity

The diameters of cleared zone(mm)	[△Leu^3^]surfactin	[△Asp^5^]surfactin	[△Leu^6^]surfactin	Surfactin
Hemolysis rings	8.29 ± 0.28	11.75 ± 0.37	7.87 ± 0.17	11.62 ± 0.45
*B. pumilus*	17.14 ± 0.16	19.71 ± 0.85	19.17 ± 0.16	19.36 ± 0.23
*F. moniliforme*	10.68 ± 0.50	7.04 ± 0.22	13.12 ± 0.49	7.09 ± 0.15

As it can be seen from Table 4, a molecular recombinant method was employed to alter the surfactin gene cluster, and the properties of the resulting novel surfactins were found to be altered, after production by fermentation. [∆Leu^6^]surfactin exhibited the ability to inhibit fungi, and the MIC of [∆Asp^5^]surfactin was higher than surfactin A. The MIC of [∆Leu^6^]surfactin for F. *moniliforme* was 50 μg/mL. These results revealed that the lack of leucine in surfactin reduced surfactin hemolytic activity while retaining antibacterial activity. At the same time, hemolytic and antibacterial activities were increased because of the lack of aspartic acid in surfactin.

**Table 4:** The minimum inhibitory concentrations (MICs) of lipopeptide antibiotics produced by *Bacillus subtilis*

Indicator strain	MIC (μg/mL)			
	[∆Leu^3^]surfactin	[∆Asp^5^]surfactin	[∆Leu^6^]surfactin	SurfactinA
*Bacillus cereus* AS1.1846	50	25	50	100
*Staphylococcus aureus* AS1.2465	50	25	50	50
*Micrococcus luteus* CMCC28000	200	50	400	200
*Pseudomonas fluorescens* AS1.1802	600	400	500	400
*Salmonella enteritidis* CICC21527	400	200	300	200
*Bacillus subtilis* ATCC9943	50	25	50	100
*Fusarium moniliforme* ATCC3893	200	ND	50	ND

ND means no detected of the minimum inhibitory concentrations for indicator strain

### Inhibitory ability of [∆Leu^6^]surfactin for *F. moniliforme* hyphae and spores

The results shown in Table 5 revealed that [∆Leu^6^]surfactin’s inhibitory ability for *F. moniliforme* hyphae and spores increased was concentration dependent. [∆Leu^6^]surfactin at 6.25 μg/mL exhibited 20.23 and 16.27 % (both *p <* 0.01) inhibitory ability for hyphae and spores, respectively. However, 50 μg/mL of [∆Leu^6^]surfactin showed stronger inhibitory ability (55.15 and 64.45 %, respectively). [∆Leu^6^]surfactin at 400 μg/mL completely inhibited hypha and spore growth. Thus, [∆Leu^6^]surfactin significantly inhibited *F. moniliforme* growth.

**Table 5:** The inhibitory ability of [∆Leu^6^]surfactin for hyphae and sporulation of *Fusarium moniliforme*

[∆Leu^6^]surfactin concentration μg/mL	Colony diameter (mm)	Inhibition of hyphae growth (%)	The number of spores (×10^6^/ml)	Inhibition of spores (%)
CK	39.58 ± 0.35^a^	0	30.1 ± 2^a^	0
6.25	31.57 ± 0.22^a^	20.23	25.2 ± 1.5^a^	16.27
12.5	27.65 ± 0.13^b^	30.14	22.5 ± 1^b^	25.24
25	21.67 ± 0.36^c^	45.25	20.3 ± 0.6^c^	32.55
50	17.75 ± 0.61^d^	55.15	10.7 ± 0.2^d^	64.45
100	9.83 ± 0.37^e^	75.16	3.5 ± 0.5^e^	88.37
200	1.63 ± 0.07^f^	95.88	0.5 ± 0^f^	98.33
400	ND	100	ND	100

ND means no detected of hyphae or sporulation for *Fusarium moniliforme*. a, b, c, d, e, f letters indicated significant differences (*P* value < 0.01)

### Influence of *F. moniliforme* hyphae by [∆Leu^6^]surfactin

**Fig. 5:**
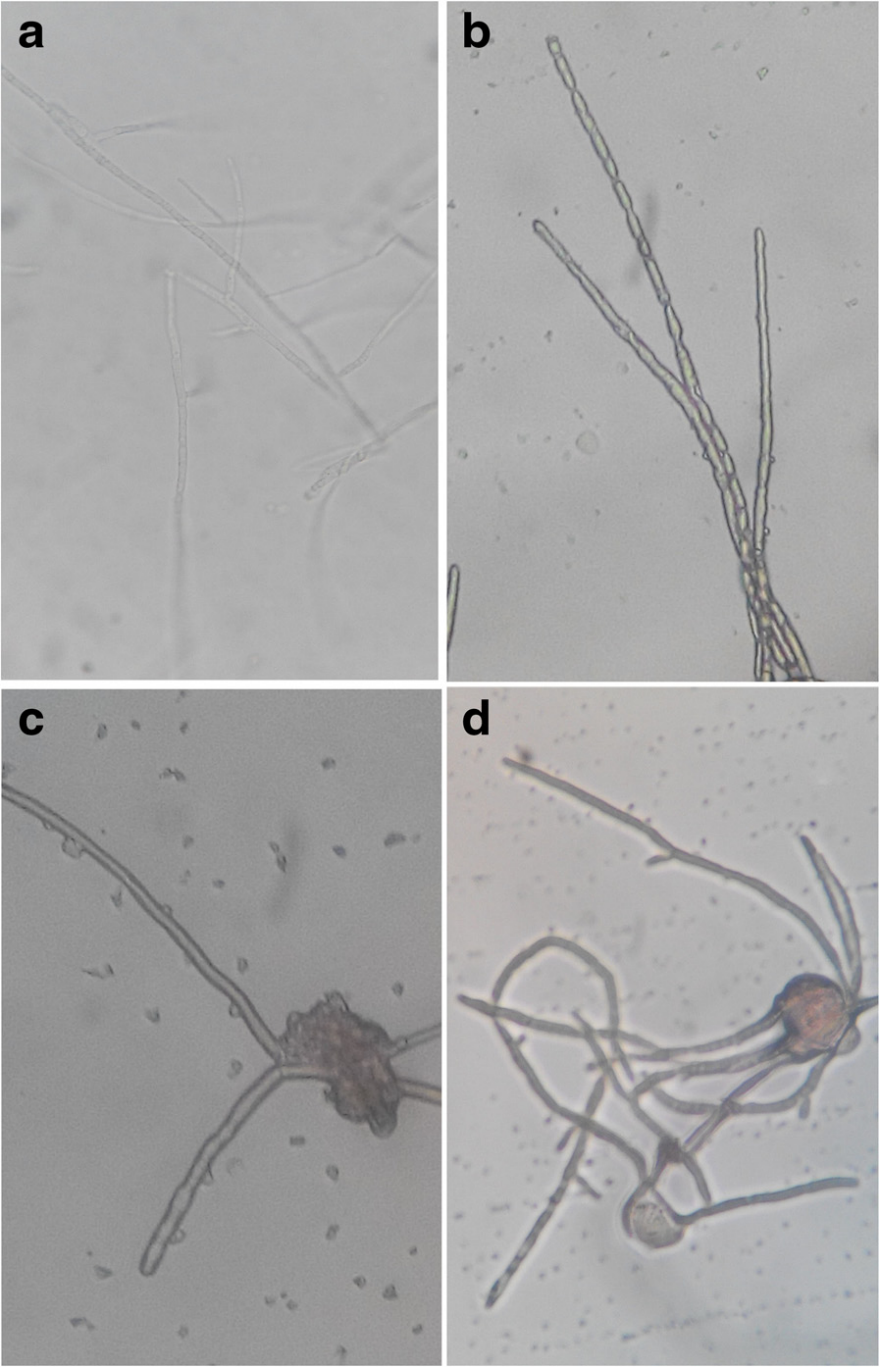
800 × micrographs of optical microscope for hyphe of *Fusarium moniliforme* treated by [∆Leu^6^]surfactin. **a** the hypha treated without [∆Leu^6^]surfactin; (**b**) the hypha treated with 50 μg/mL of [∆Leu^6^]surfactin for 1 h; (**c**) the hypha treated with 50 μg/mL of [∆Leu^6^]surfactin for 2 h; (**d**) the hypha treated with 50 μg/mL of [∆Leu^6^]surfactin for 4 h

**Fig. 6:**
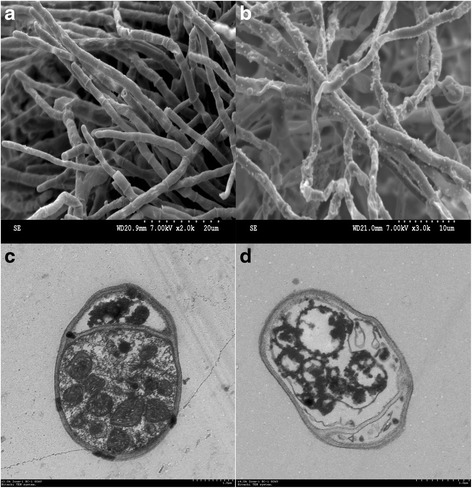
SEM and TEM micrographs of hyphae of *Fusarium moniliforme* treated by [△Leu^6^]surfactin. **a**, the hyphae treated without [△Leu^6^]surfactin (SEM); **b**, the hyphae treated with 50 μg/mL of [△Leu^6^]surfactin (SEM); **c**, the hyphae treated without [△Leu^6^]surfactin (TEM); **d**, the hyphae treated with 50 μg/mL of [△Leu^6^]surfactin (TEM)

Compared with control hyphae, many small vesicles were observed on numerous mycelia after treatment with 50 μg/mL [∆Leu^6^]surfactin for 1 h (Fig. 5b); there were no observable vesicles in control hyphae. After treatment for 2 h, vesicles on mycelia became large and a relatively large projections emerged in the central regions of mycelia. After treatment for 4 h (Fig. 5d), the abnormal central mycelial enlargements were ruptured and mycelia appeared as thin strips. This phenomenon was not observed in controls (Fig. 6a).

*F. moniliforme* hyphal structures were observed by SEM and TEM (Fig. 6). Hyphae treated without [∆Leu^6^]surfactin grew normally with a straight, smooth appearance (Fig. 6a), while bending and rough structures were observed after treatment with 50 μg/mL of [∆Leu^6^]surfactin (Fig. 6b). TEM images of growing, healthy, and normal hyphae, treated without [∆Leu^6^]surfactin, showed smooth surfaces and all cellular organelles were visible and in normal arrangements (Fig. 6c). In contrast, although hyphal structures remained intact when treated with 50 μg/mL [∆Leu^6^]surfactin, organelles were gathered in clumps and some large vacuoles were noticeable in their central regions (Fig. 6d). SEM and TEM observations indicated that [∆Leu^6^]surfactin clearly affected *F. moniliforme* growth.

### Impact of [∆Leu^6^]surfactin on *F. moniliforme* spores

**Fig. 7:**
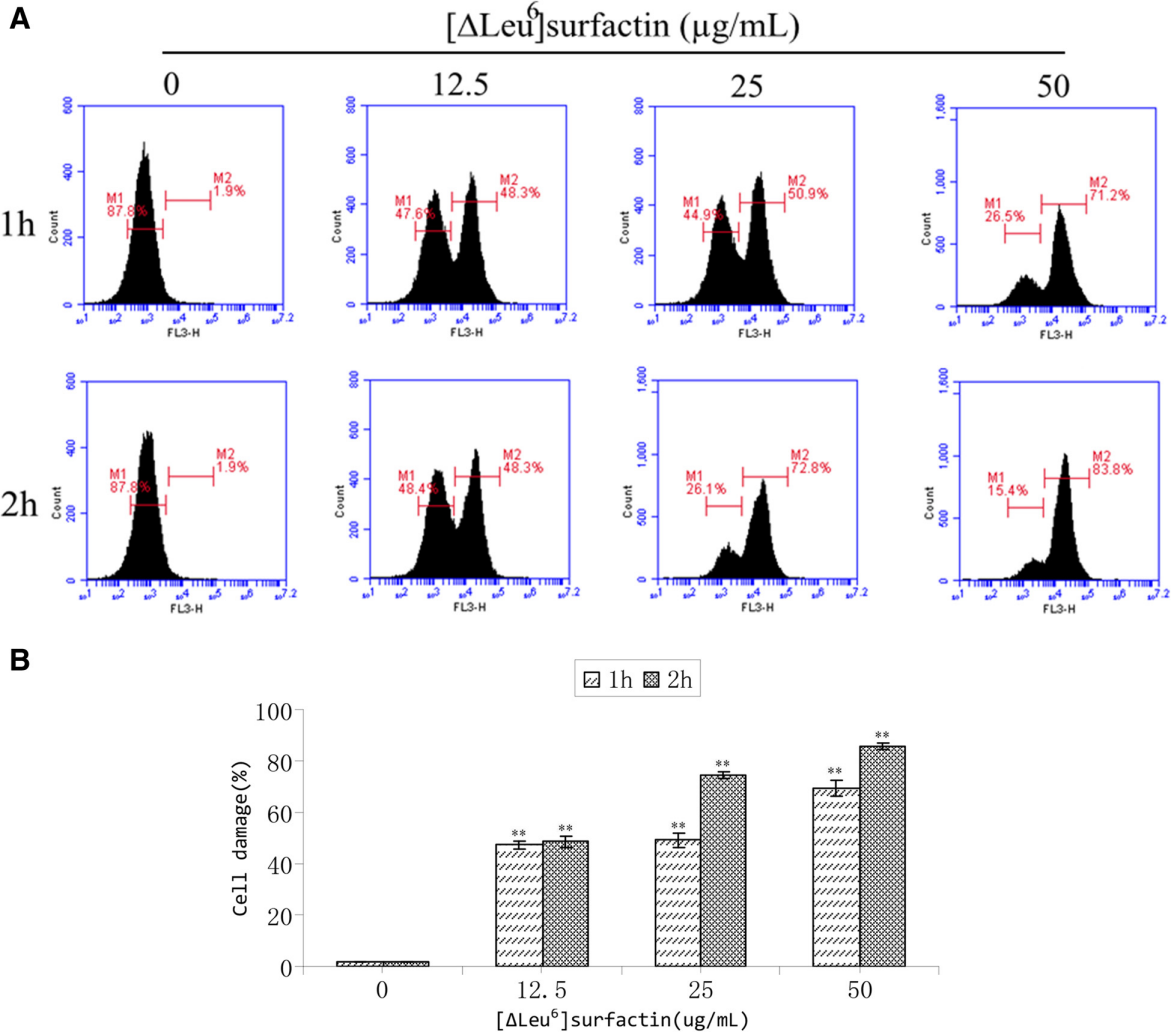
cell damage of *Fusarium moniliforme* spores by flow cytometry. **a**. flow cytometry graphs of *Fusarium moniliforme* spores treated by 0, 12.5, 25, 50 μg/mL of [∆Leu^6^]surfactin. M1 and M2, events of *Fusarium moniliforme* spores dead cells, 10 000 cells were detected. **b**. Statistical analysis of [∆Leu^6^]surfactin for cell damage of *Fusarium moniliforme* spores, Asterisk letters indicated significant differences between control group and treatment group. (*P value < 0.05, **P value < 0.01).

In samples treated for 1 h with a final concentration of 12.5 μg/mL [∆Leu^6^]surfactin, a portion of *F.* spores were already stained with fluorescent dye (Fig. 7). With increased surfactin concentration, fluorescent dye staining rapidly increased. Two-hour samples treated with the same concentration did not show increased fluorescent dye spore staining. However, in 25 and 50 μg/mL [∆Leu^6^]surfactin treated samples, staining of spores increased significantly. The increase in the number of spores was determined by the intrusion of fluorescent dyes. Thus, these observations revealed that [∆Leu^6^]surfactin’s effects on F. *moniliforme* spores also led to damage or apoptosis of spores.

### Impact of [∆Leu^6^]surfactin on the integrity of *F. moniliforme* mycelia

The effects of a surfactin on mycelium integrity can be reflected in the release of intracellular contents, such as an increased release of nucleic acid and proteins (assessed by changes in OD_260_ and OD_280_, respectively).

**Fig. 8:**
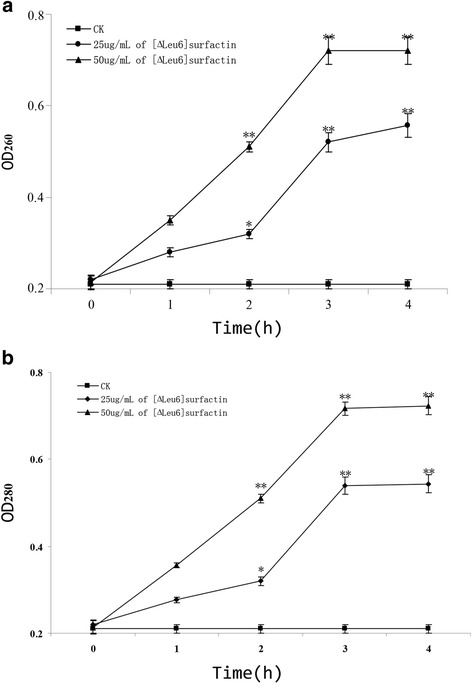
Divulgation of nucleic acids and proteins of *Fusarium moniliforme*. CK, samples treated without surfactin. Divulgation of nucleic acids (OD_260_) (**a**) and proteins (OD_280_) (**b**) were calculated (*P value < 0.05, **P value < 0.01). The concentration of [∆Leu^6^]surfactin was 25 and 50 μg/mL. The time of treatment reached 3 hours

After treatment of *F. moniliforme* with 25 and 50 μg/mL of [∆Leu^6^]surfactin, *F.* cell nucleic acids and proteins were rapidly released into the external bacterial body, detected by spectroscopic absorption analysis of released nucleic acids and proteins (Fig. 8). The OD_260_ and OD_280_ of extracellular fluids increased rapidly in samples treated for 2 h with 50 μg/mL [∆Leu^6^]surfactin. After 3 h of treatment, the OD_260_ and OD_280_ reached their maxima and ceased to increase.

### DNA binding assay

**Fig. 9:**
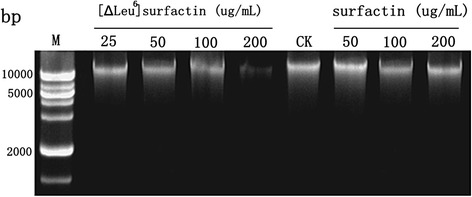
DNA binding assay by [∆Leu^6^]surfactin and surfactin. CK, *Fusarium moniliforme* DNA was mixed with PBS as control. *Fusarium moniliforme* DNA was mixed with different amounts of surfactin and [∆Leu^6^]surfactin, and then the reaction mixtures after incubating for 1 h at room temperature were performed to 1 % agarose gel electrophoresis

With increased antimicrobial lipopeptide concentrations, the binding capacity of *F. moniliforme* DNA gradually strengthened, indicating that [∆Leu^6^]surfactin imposed a certain effect on the fungal genome. As shown in Fig. 9, treatment of fungal samples with 200 μg/mL [∆Leu^6^]surfactin resulted in the *Fusarium* genome DNA showing some dispersion. These results showed that surfactin possessed the ability to bind to fungal DNA, with [∆Leu^6^]surfactin exhibiting the greatest such ability.

## Discussion

In this study, a method for marker-free knockout of surfactin synthase was adopted and surfactin synthase successfully altered in three different modules. These modules were responsible for the synthesis of leucine and aspartic acid. Some deletion module surfactin derivatives have already been demonstrated, including versions in which the second and last modules of surfactin synthetase were deleted [32, 33]. Schneider et al. have previously reported a different modification method, which relied on molecular surgery within the domains to achieve an exchange of A- units [22]. This approach has been successfully applied to position 7 in surfactin NRPS [34]. From previous reports, the module containing the epimerization domain has never before been removed. The comS regulation factor is the key factor in forming competent B. *subtilis* cells. As the comS regulatory factor is contained in the aspartate module, it is difficult to delete this module. Here, it is reported for the first time the complete deletion of this module containing this regulatory factor.

The purification method for novel surfactins draws on experience with a former method for surfactin extraction and purification [35]. For detection of biological activity, the hemolytic activity of [∆Asp^5^]surfactin was found to have strengthened antimicrobial activity. For filamentous fungi, no inhibition zones have been discovered. These results revealed that this method for structural transformation of surfactin was feasible and practical.

However, biological activity tests for novel surfactins lacking *D*-leucine showed that the hemolytic activities of [∆Leu^3^]surfactin and [∆Leu^6^]surfactin were significantly decreased compared with the original surfactin A. [∆Leu^6^]surfactin drastically inhibited the growth of *F. moniliforme* hyphae and spores when the [∆Leu^6^]surfactin was at 50 μg/mL. Therefore, it was evident that [∆Leu^6^]surfactin significantly inhibited *F. moniliforme* growth. Surfactin is a well-known lipopeptide biosurfactant with antimicrobial activity, but there is little knowledge regarding surfactin’s antifungal activity [36]. Surfactins are not alone in inhibiting filamentous fungi, but C15 surfactin and antifungal drugs have been reported to have a synergistic effect. Surfactins have two polar amino acid residues, such as Glu and Asp, and have been concluded to bind with DNA via hydrogen bonds [37, 38].

SEM and TEM observations indicated that [∆Leu^6^]surfactin obviously affected *F. moniliforme* growth by causing morphological changes in hyphae, suggesting that [∆Leu^6^]surfactin markedly contributed to inhibiting fungal growth. DNA binding results indicated that [∆Leu^6^]surfactin negatively influenced the maintenance of DNA integrity by binding to *F. moniliforme* DNA, which might in turn genetically affect DNA function for *F. moniliforme* growth.

This provides the possibility for a new surfactin with potential for food industry applications. As the original surfactin A has relatively strong hemolytic activity, its use has been restricted in the food industry. The purpose of this study was to develop a novel class of antibacterial lipopeptides from surfactin that possessed reduced cytotoxicity with no significant reduction in antimicrobial activity.

The inhibitory ability of filamentous fungi by three novel surfactins was examined here. Very interestingly, [∆Leu^3^]surfactin and [∆Leu^6^]surfactin exhibited the ability to inhibit filamentous fungi. These findings extended the known properties of surfactin derivatives as well as related changes in antifungal activity. Further research is needed into the mechanism of this antifungal inhibition.

## Conclusion

In this study, molecular biological tools were successfully employed to transform surfactin synthase and produce three new substances, [∆Leu^3^]surfactin, [∆Asp^5^]surfactin, and [∆Leu^6^]surfactin. Analyses of these substances’ biological activity showed that [∆Leu^3^]surfactin and [∆Leu^6^]surfactin possessed significantly reduced hemolytic activity but with the concurrent appearance of inhibitory ability for filamentous fungi.

## Abbreviations

CK: control check
CFU: colony-forming unit
DNA: deoxyribonucleic acid
ERM: erythromycin
ELISA: enzyme-linked immuno sorbent assay
FTICR-MS: fourier transform ion cyclotron resonance mass spectrometry
HPLC: high performance liquid chromatography
LB: Luria broth
LC: liquid chromatography
MIC: minimal inhibitory concentration
NRPS: non-ribosomal peptide synthetases
OD: optical density
PBS: phosphate buffered saline
PCR: polymerase chain reaction
PDA: potato dextrose agar
PI: propidium iodide
SD: standard deviation
SOE: special operations executive
SrfA: surfactin A
SEM: scanning electron microscope
TEM: transmission electron microscope
